# Switching intention toward conversational AI for health information seeking: a push–pull–mooring analysis

**DOI:** 10.3389/fpsyg.2026.1877044

**Published:** 2026-10-19

**Authors:** Zhaoqi Zhou, Yan Lin, Quanzhi Chen, Yanting Wu, Yimingzi Chen, Ke Zhang, Jing Wang

**Affiliations:** 1 School of Communication, Soochow University, Suzhou, Jiangsu, China; 2 Ulink High School of Suzhou Industrial Park, Suzhou, Jiangsu, China; 3 City Culture and Communication College, Suzhou City University, Suzhou, Jiangsu, China

**Keywords:** fsQCA, health information seeking, medical AI resistance bias, PPM model, switching intention

## Abstract

**Introduction:**

With the rapid development of artificial intelligence, users are increasingly turning to conversational AI for health information seeking. This study applies the Push-Pull-Mooring (PPM) framework to explain users' switching intention from social media to conversational AI for comparable health-information-seeking tasks.

**Methods:**

Survey data were collected from 2,876 Chinese respondents. Structural equation modeling (SEM) and fuzzy-set qualitative comparative analysis (fsQCA) were used to examine the associations of push, pull, and mooring factors with switching intention.

**Results:**

Health information overload and cyberchondria were positively associated with dissatisfaction with social media, which was positively associated with switching intention. Perceived personalization and perceived anonymity were positively associated with flow experience during conversational AI use, which was also positively associated with switching intention. In contrast, medical AI resistance bias and switching costs were negatively associated with switching intention. The fsQCA results further showed that high switching intention was more likely to emerge under configurations characterized by strong pull and weak mooring conditions, whereas low switching intention was typically associated with high mooring conditions.

**Discussion:**

The findings highlight the joint roles of push, pull, and mooring factors in shaping users' switching intention and demonstrate that switching intention can arise through multiple asymmetric configurations.

## 1 Introduction

Online health information seeking has become an important way for the public to obtain health knowledge. With the rapid development of generative artificial intelligence (AI), particularly large language models (LLMs) such as ChatGPT, DeepSeek, and Gemini, users' media habits are undergoing significant changes. Unlike keyword-based retrieval on social media or search engines, conversational AI allows users to obtain health information through interactive natural-language dialogue (Peng et al., 2025). Prior research suggests that switching from existing information channels to generative AI is a meaningful research problem. In health contexts, users have been found to develop intentions to shift from traditional health-information platforms to generative AI (Qiu et al., 2025), while adjacent studies show migration from social Q&A communities to generative AI for comparable information-seeking tasks (Zhou and Wu, 2024). These studies suggest that although social media and conversational AI are not identical platforms, they may function as competing candidate channels for comparable health information-seeking tasks.

Existing research focuses on how the costs and benefits in AI-driven search behavior influence users' actual willingness to use AI for searching (Pham et al., 2024), showing that the higher users' self-efficacy with ChatGPT, the more likely they are to proactively use AI to search for information (Mun, 2025). Drawing on the S-O-R theoretical framework, Zhou and Ma (2025) propose that content quality and system quality influence users' intention to continue using the technology. From a user-centered perspective, whether through selective retrieval or intelligent generation, any approach that satisfies users' information needs can essentially be regarded as a form of information-seeking behavior. Overall, conversational AI is reshaping health information seeking by providing an alternative to conventional searching and filtering through dialogue-based information generation.

However, existing research still has limitations in two respects. First, the literature on health information seeking has largely focused on contexts such as search engines or social media, while little attention has been paid to AI-enabled health information seeking. Second, most research on AI adopts a technology acceptance perspective and rarely examines how switching intentions are formed when users consider shifting their primary reliance from existing information channels to conversational AI. As a result, there is a lack of a systematic theoretical framework to explain how users shift toward conversational AI for health information seeking. We introduce the push–pull–mooring (PPM) model and construct a conceptual framework to examine users' switching intention from social media to AI in the context of health information seeking. This study aims to address the following three questions:

Q1: In the context of health information seeking, which push, pull, and mooring factors are associated with users' intention to shift their primary reliance from social media to conversational AI?
Q2: How do mooring factors moderate the associations between push and pull factors and switching intention?
Q3: Which factors play a central role in shaping individuals' intention to shift their primary reliance for health information seeking?

## 2 Theoretical framework and hypotheses development

### 2.1 Health information seeking

Health Information Seeking Behavior (HISB) refers to the information-related behaviors exhibited by individuals in the process of seeking and verifying health-related knowledge (Manafo and Wong, 2012). With the rapid development of social media, obtaining health information through social media has attracted increasing scholarly attention. Social media has become an important venue for consumer health information seeking, offering both informational and social benefits while also introducing concerns about accuracy and quality (Zhao and Zhang, 2017). Neely et al. (2021) found that during the COVID-19 pandemic, despite the low accuracy of COVID-19 related information on social media, people still relied heavily on social media to search for such information in order to obtain emotional support and reduce internal uncertainty. However, across various health topics such as vaccination, cancer, and mental health, misinformation is highly prevalent on social media, making it difficult for ordinary users to discern its accuracy (Kbaier et al., 2024). Social media has become a prominent channel for health information seeking because it offers diverse information, peer experiences, and emotional support. However, negative experiences such as misinformation and information overload have become increasingly prominent. These factors may reduce users' satisfaction with seeking health information on social media and motivate them to explore alternative information channels, thereby serving as potential “push” factors within the PPM framework.

Conversational AI refers to AI systems that interact with users through ongoing natural-language dialogue and generate responses based on their expressed needs (Sun et al., 2024). AI-enabled conversational health information seeking refers to the use of such systems to obtain and understand health-related information. In this study, conversational AI is conceptualized as an alternative channel for accessing and interpreting health information, rather than as a provider of diagnosis or treatment. With the development of generative AI, researchers have attempted to train conversational systems using large-scale community health question and answer datasets, thereby enabling “conversational health information retrieval” (Wong et al., 2011). Recent task-specific studies have reported favorable evaluations of conversational AI in terms of response accuracy, appropriateness, empathy, structure, and supporting documentation under particular models, prompts, and clinical topics (Harding et al., 2025; Chen D. et al., 2024; Ghanem et al., 2024; Collins et al., 2025). However, such findings do not establish that conversational AI is inherently more accurate, reliable, or clinically beneficial than social media, professional care, or other information sources. Its performance is context dependent, and LLMs may still produce inaccurate or misleading health information (Denecke et al., 2024).

### 2.2 The PPM model

The push–pull–mooring (PPM) model posits that certain negative factors “push” people away from their original location, while positive factors “pull” them toward a new destination (Bansal et al., 2005). Moon (1995) introduced “mooring factors” to the model to explain the situational or individual constraints that hinder or facilitate migration decisions.

The PPM model has been widely used to explain user migration and discontinuous use in marketing and information systems contexts, such as online communities and social media. Hwang et al. (2018) applied the PPM model to user migration in social networking service (SNS), introducing the concept of SNS fatigue as a push factor, and found that social interaction overload and non-volitional relationships (push factors), as well as alternative attractiveness and group pressure (pull factors), significantly influence users' switching intentions. Li et al. (2025), drawing on the PPM model, developed and empirically tested users' switching intentions between two alternative information seeking modes, namely human-human and human-machine, and revealed the roles of variables such as privacy risk, interactivity, anthropomorphism, and social support in information search. In the field of health communication, the PPM model can also be used to examine users' intentions to shift between alternative channels for health information seeking. Lai et al. (2021) integrated the PPM model with “status quo bias” to explain users' switching intention toward medical AI chatbots. Qiu et al. (2025) Using PPM model to explore health information switching intentions in the context of generative artificial intelligence. Extending the PPM framework beyond single-technology adoption, this study examines users' intended shifts in primary reliance between social media and conversational AI as alternative health-information-seeking channels.

In summary, the PPM model provides a structured framework for understanding switching decisions involving multiple platforms and technological alternatives. In this study, switching intention refers to users' prospective self-reported willingness to rely more on conversational AI than on social media for comparable health-information-seeking tasks. It is distinct from actual switching, which would require an observable change in users' subsequent channel-use behavior. Accordingly, a high switching intention does not necessarily mean that users have abandoned social media or have actually changed their primary information source.

### 2.3 Push factors

The information ecology and content quality of social media may prompt users to seek new media for health information searching. Health information overload refers to a state in which the amount of health information encountered on social media exceeds individuals' processing capacity (Chen et al., 2022). While social media is shaping a new public health information environment, it also exposes users to large amounts of contradictory health information (Kim et al., 2007), and perceived information overload has a significant positive effect on fatigue and dissatisfaction with social media services.

Meanwhile, social media users may experience cyberchondria, which refers to excessive health anxiety associated with repeated or excessive online health-information searching (Vismara et al., 2020). Ding et al. (2025) found that in online health information consultations, cyberchondria leads users to intermittent discontinuance. The anxiety triggered by users' cyberchondria is likely to be attributed to the social media they rely on, thereby increasing their dissatisfaction with social media. Thus, we propose:

**H1**: Health information overload (H1a) and cyberchondria (H1b)are positively associated social media dissatisfaction.

Satisfaction reflects users' overall evaluation of their recent and past usage experiences (Bhattacherjee, 2001). Multiple studies on online platforms and social media have demonstrated that dissatisfaction is a key factor driving users to switch (Sekarputri et al., 2024). Social media fatigue and dissatisfaction both significantly and positively influence users' intention to discontinue using social media (Zhang et al., 2016). Dissatisfaction refers to users' negative overall evaluation of their health-information-seeking experiences on social media, including perceived failure to meet their informational and experiential expectations. Therefore, when users feel dissatisfied with health information on social media, they may report a stronger intention to shift their primary reliance toward conversational AI for health information seeking. We propose:

**H2**: Social media dissatisfaction is positively associated with switching intention.

### 2.4 Pull factors

AI as an emerging tool for health information seeking exhibits many features that are attractive to users. Personalization is an important factor driving AI use (Baek and Kim, 2023). Perceived personalization refers to the ability of AI-generated content (AIGC) to produce tailored information based on users' needs and to deliver increasingly precise services through ongoing interactions (Zhou et al., 2025). When users interact with generative AI, personalized features can make them feel valued, which has a significant positive effect on their flow experience (Zhou and Li, 2025). Ghani and Deshpande (1994) defined flow experience as an immersive state of intense concentration and psychological enjoyment that users experience while interacting with a system. Such flow experience enhances users' sense of exploration and their willingness to use the system.

Perceived anonymity refers to the extent to which users believe that their identities remain unidentifiable when interacting with AI (Chen et al., 2019). In the context of AI-enabled health information seeking, users' subjective assessment of how well their personal information is protected constitutes perceived anonymity. Especially for highly stigmatized health issues such as sexually transmitted diseases and mental health problems, AI tends to be more acceptable to users (Miles et al., 2021), who are more willing to express their thoughts and become more focused on the interaction process. When searching for information via AI, anonymity can also help users receive better emotional support, thereby encouraging more active interaction with AI (Gan, 2025). Therefore, perceived personalization and perceived anonymity can make users more focused and immersed in the interaction process, leading to a stronger flow experience. Thus, we hypothesize:

**H3**: Perceived personalization (H3a) and perceived anonymity (H3b) are positively associated with flow experience.

The flow experience is a powerful positive psychological driver that motivates users to continue using a system (Hong and Xu, 2017). In research based on the PPM model, flow experience can be conceptualized as a proximal pull factor associated with stronger switching intention. Zhou (2015) found that flow experience can attract users to adopt and use online payment services. Zhou and Wu (2024) found that flow experience was positively associated with users' intention to shift from social Q&A communities to generative AI. Therefore, in conversational health information seeking, users may also experience a strong sense of flow experience through interaction with AI, thereby strengthening their switching intention. Thus, we hypothesize:

**H4**: Flow experience is positively associated with switching intention.

### 2.5 Mooring factors

Lai et al. (2021) integrated the PPM model with status quo bias, arguing that factors such as habit, risk perception, and uncertainty are associated with a stronger preference for existing information-seeking methods and a weaker intention to adopt medical AI chatbots as an alternative channel. Chen T. et al. (2024) found in their research on new technology adoption that switching costs act as mooring factors. Accordingly, this study treats resistance to medical AI and switching costs as two mooring factors influencing users' switching intentions.

Longoni et al. (2019) pointed out that resistance to medical AI refers to individuals' lower levels of compliance and trust when facing diagnoses or health recommendations provided by AI systems, even when the AI's recommendations are objectively superior. Medical AI resistance bias (AIRB) refers to users' attitudinal tendency to distrust or resist relying on AI-generated health information and recommendations because of concerns about AI's accountability, contextual judgment, and lack of human relational care. Gaczek et al. (2023) further found that health anxiety significantly influenced patients' acceptance of AI health information. Disease uncertainty refers to an individual's inability to determine illness-related information and to understand their condition and subsequent treatment during medical consultations (Mishel, 1988). Huang and Shen (2018) proposed the concept of health condition uncertainty, which emphasizes the uncertainty that arises when laypeople become doubtful about their own health status. It refers to individuals' perceived uncertainty about their bodies and health issues in everyday life. To reduce this form of health condition uncertainty, individuals typically actively seek information that aligns with their existing cognitions (Jin et al., 2025).

Huang and Tan (2024) argue that patients' conservatism toward and resistance to AI are related to their emotional dependence on physicians and their humanistic expectations within the relationships with doctors. Wang and Liao (2025) argue that In health-related contexts, trust is closely tied to accountability, which is still primarily provided by physicians. Therefore, a strong doctor–patient relationship also makes individuals more likely to perceive AI as a potential threat. Such resistance may discourage users from shifting toward conversational AI and is therefore conceptualized as a mooring factor. Thus, we propose:

**H5**: Health condition uncertainty (H5a) and relationships with doctors (H5b) are positively associated with medical AI resistance bias.

Existing research indicates that even when AI offers treatment recommendations identical to those of human physicians, trust in the former remains significantly lower (Wu et al., 2022). When users hold skeptical, worried, or even resistant attitudes toward AI, their switching intention may still be weakened even if they are dissatisfied with social media. Compared with search engines and health websites, users tend to be more cautious about health advice generated by AI (Yun and Bickmore, 2025). Therefore, we propose:

**H6**: Medical AI resistance bias is negatively associated with switching intention.
**H7**: Medical AI resistance bias negatively moderates the association between dissatisfaction and switching intention.

Loss aversion leads individuals to weight potential losses more heavily than potential gains, thereby overestimating the time, effort, and adaptation costs that may arise from switching information-seeking channels (Kahneman et al., 1991). Gong et al. (2020) found that loss aversion amplifies individuals' subjective perceptions of switching costs in digital service migration. Switching information-seeking channels not only entails objective costs in terms of time and adaptation, but also represents a psychological loss associated with changes in usage habits. Loss aversion makes users particularly concerned that the new service might perform worse, leading to a perceived loss of the efficiency they previously enjoyed (Gong et al., 2020).

Performance risk refers to the risk that individuals perceive that a new technology or service may fail to achieve its expected functions or outcomes. It can alter users‘ subjective evaluations of overall costs by influencing their assessments of time risk, learning risk, and outcome risk (Featherman and Pavlou, 2003). Liu et al. (2025) found that performance risk is a key inhibiting factor in users' adoption of new technologies. When users worry that AI-based health information seeking may be unstable in terms of usability, they tend to overestimate the associated risks. Therefore, as users shift to conversational health information seeking, both uncertainty about the technology's utility and loss aversion during the switching process increase their perceived switching costs. Therefore, we propose:

**H8**: Loss aversion (H8a) and performance risk (H8b) are negatively associated with switching costs.

Switching costs refer to the various costs incurred by users when switching between different services (Chang et al., 2017). In the PPM literature, switching costs are commonly regarded as mooring factors that hinder user migration (Liu et al., 2021). Cheng (2025) found that users' switching behavior between intelligent personal assistant devices is influenced by switching costs. When users perceive cross-platform switching costs to be too high, they may report weaker switching intentions and a stronger preference for maintaining their reliance on the incumbent platform. In addition, mooring factors play a moderating role in users' migration intentions (Chang et al., 2017). Specifically, switching costs not only directly weaken users' switching intention but also attenuate the positive effects of favorable experiences on switching intention. When users perceive high switching costs, even if they experience a strong sense of flow during trial use, concerns about potential losses may prevent favorable experiences from developing into a strong switching intention. Therefore, we propose:

**H9**: Switching costs are negatively associated with switching intention.
**H10**: Switching costs negatively moderate the association between flow experience and switching intention.

The conceptual framework is shown in Figure 1.

**Figure 1:**
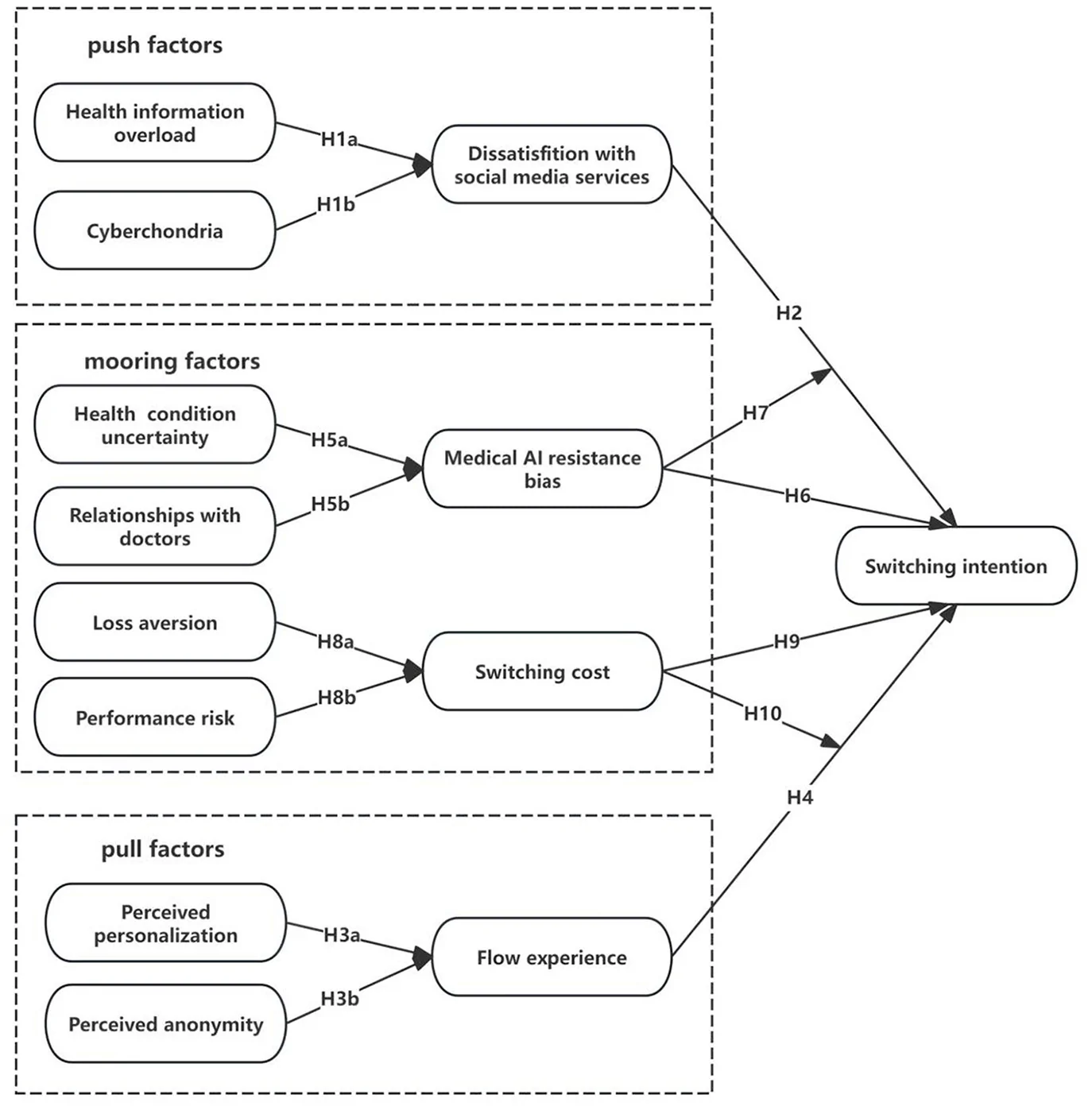
A PPM-Based Mogdel of users' switching intention from social media to conversational AI for health information seeking.

## 3 Research methods

### 3.1 Measurement scales

This study adopts a questionnaire survey method. The questionnaire was designed based on the research model and measured using a five-point Likert scale. The demographic information of the survey includes gender, age, income level, educational attainment, place of residence, health insurance status, health condition, frequency of AI use, and experience with online health consultation. The measurement items of all variables are presented in Table 1.

**Table 1:** Measurement items.

Variables	Items	Measurements	Sources
Health information overload (HIO)	HIO1	The large amount of decision-relevant health information on social media makes it difficult for me to choose.	Zhang et al., 2026
	HIO2	I am often distracted by too much decision-relevant health information on social media.	
	HIO3	The large amount of health information on social media makes me feel mentally fatigued.	
Cyberchondria (CYB)	CYB1	After searching for health information, I worry that I might have a disease.	Jokić-Begić et al., 2019
	CYB2	After searching for health information, I feel depressed.	
	CYB3	After searching for health information, I feel confused about the information I found.	
	CYB4	Once I start searching for health information, it is difficult to stop.	
Dissatisfaction with social media services (DSSM)	DSSM1	I feel dissatisfied with my overall experience using social media to seek health information.	Zhang et al., 2016
	DSSM2	I feel displeased about my overall experience using social media for seeking health information.	
	DSSM3	I feel discontented about my overall experience using social media for seeking health information.	
	DSSM4	I am not delighted with my overall experience using social media to seek health information.	
Health Condition Uncertainty (HU)	HU1	I am not sure whether or not I am sick.	Huang and Shen, 2018
	HU2	I am not sure what happened to me regarding my illness.	
	HU3	I am not sure where I can find the resources or support to help me.	
	HU4	I am not sure whether I should feel positive or negative about my current situation.	
	HU5	I am not sure how likely my symptom/illness will get worse.	
Relationships with doctors (RWD)	RWD1	I let my doctor handle the details of my health.	Lai et al., 2021
	RWD2	Doctors are my most trusted source of health information.	
	RWD3	When I have a health concern, my first step is to contact a doctor.	
Loss aversion (LA)	LA1	I feel safer using healthcare services that I am familiar with.	
	LA2	I prefer to keep using familiar healthcare services to trying out new ones.	
	LA3	When considering obtaining healthcare services, I prefer to use well-established healthcare services.	
	LA4	Whenever I need to have a health consultation, I unconsciously stare at the services I am familiar with.	
Performance risk (PR)	PR1	I am worried that AI might not understand patients' needs.	
	PR2	There is a possibility of malfunction and performance failure, so AI might fail to deliver accurate diagnoses or recommendations.	
	PR3	I am concerned about the accuracy and precision of suggestions provided by AI.	
Medical AI Resistance Bias (AIRB)	AIRB1	I would choose to rely more on human doctors rather than AI.	
	AIRB2	I prefer to communicate with human doctors rather than AI.	
	AIRB3	I might regret using medical AI services because they may waste my time.	
Switching Costs (SC)	SC1	I believe that learning to use AI requires a considerable amount of time and effort.	Li et al., 2025
	SC2	I believe that signing up to use a new AI is inconvenient.	
	SC3	I have already invested a considerable amount of time and effort in learning and using AI	
Perceived Personalization (PP)	PP1	AI understands my health needs.	Qin et al., 2024
	PP2	AI takes my health needs as its own preferences.	
Perceived anonymity (PA)	PA1	People cannot identify true-me from my messages in AI.	Jung et al., 2012
	PA2	People cannot locate me in real life based on my information and messages in AI.	
	PA3	I cannot recognize the users of AI when we are passing by in real life.	
	PA4	I can control the level of my anonymity in AI.	
Flow experience (FE)	FE1	AI allowed me to control the interaction.	Zhou et al., 2025
	FE2	When using AI, I was totally absorbed in the activity.	
	FE3	Using AI aroused my curiosity.	
	FE4	Using AI was intrinsically interesting.	
Switching Intention (SI)	SI1	I am considering switching from social media to AI platforms.	Qiu et al., 2025
	SI2	I intend to switch from social media to AI platforms.	
	SI3	When AI platforms' functionality is satisfactory, I will replace social media with AI platforms.	

All items were measured on a five-point Likert scale ranging from 1 (“strongly disagree”) to 5 (“strongly agree”). SC3 was reverse-coded. “R” indicates a reverse-coded item.

### 3.2 Data collection

From September 3 to October 16, 2025, participants were recruited through Xiaohongshu, Douban, and Wenjuanxing. A purposive screening procedure retained only respondents with prior experience using both social media and conversational AI for health-information seeking. A pilot test with 120 eligible users was conducted to refine item wording, response options, and questionnaire presentation before formal data collection. A total of 3,194 questionnaires were collected. After responses with unusually short completion times, patterned responses, or internally inconsistent answers were excluded, 2,876 valid questionnaires remained, yielding a valid-response rate of 90.04%.

The demographic characteristics of the respondents are presented in Table 2. Most respondents (98.4%) also reported prior experience with online health consultation platforms. Most were covered by either urban and rural resident medical insurance or employee medical insurance, while only a small proportion reported having no health insurance. Detailed respondent characteristics are reported in Table 2.

**Table 2:** Demographic characteristics of respondents.

Measurement	Item values	Total count	Per item (%)
Gender	Male	1,437	50
	Female	1,439	50
Age	Under 18 years	405	14.1
	18–30 years	491	17.1
	31–40 years	538	18.7
	41–50 years	538	18.7
	51–60 years	486	16.9
	Over 60 years	418	14.5
Monthly income (RMB)	≤ 2,000	991	34.5
	2,001–5,000	810	28.2
	5,001–10,000	537	18.7
	10,001–20,000	287	10
	20,001–50,000	202	7
	>50,000	49	1.7
Education	HIgh school or below	974	33.9
	Junior college/associate degree	769	26.7
	Bachelor's degree	827	28.8
	Master's degree or above	306	10.6
Place of residence	Tier 1 city	557	19.4
	Tier 2 city	670	23.3
	Tier 3 or Tier 4 city	833	29
	Rural area	816	28.4
Health status	Serious health problems	175	6.1
	Chronic illness	429	14.9
	Generally healthy	2,272	79
General AI-use frequency	Daily	1,078	37.5
	Weekly, but not daily	926	32.2
	Monthly, without a fixed schedule	540	18.8
	Occasionally	328	11.4
	Rarely	4	0.1
Frequency of using AI for health information seeking	Daily	698	24.3
	Weekly, but not daily	945	32.9
	Monthly, without a fixed schedule	777	27
	Occasionally	437	15.2
	Rarely	19	0.7
PRior experience using online health consultation platforms	Yes	2,829	98.4
	No	47	1.6
Health insurance coverage	Employee medical insurance	638	22.2
	Urban and rural resident medical insurance	1,975	68.7
	New Rural Cooperative Medical Scheme	6	0.2
	Other insurance	222	7.7
	No insurance	35	1.2

## 4 Results

### 4.1 Reliability and validity

Based on the corrected item–total correlation analysis, the reverse-coded switching cost item (SC3), “I have already spent a great deal of time and effort learning and using chatbots,” had a coefficient below 0.40 and was therefore removed. After deleting this item, the Cronbach's alpha for the switching cost (SC) construct was 0.784, indicating good reliability. As shown in Table 3, all Cronbach's alpha coefficients exceeded the recommended threshold of 0.70, indicating good internal consistency. All factor loadings exceeded 0.70, composite reliability (CR) values were above 0.70, and average variance extracted (AVE) values were greater than 0.50, supporting good convergent validity. As shown in Table 4, the square root of the AVE for each construct exceeded its correlations with the other constructs, supporting satisfactory discriminant validity.

**Table 3:** Reliability and validity.

Variables	Items	Loading	CR	AVE	Alpha
Health information overload (HIO)	HIO1	0.837	0.8657	0.6823	0.866
	HIO2	0.823			
	HIO3	0.818			
CYBerchondria (CYB)	CYB1	0.8	0.8795	0.6461	0.882
	CYB2	0.803
	CYB3	0.798
	CYB4	0.814
Dissatisfaction with social media services (DSSM)	DSSM1	0.833	0.8987	0.6893	0.898
	DSSM2	0.83			
	DSSM3	0.835			
	DSSM4	0.823			
Health condition uncertainty (HU)	HU1	0.802	0.9023	0.6487	0.904
	HU2	0.814			
	HU3	0.802			
	HU4	0.804			
	HU5	0.805			
Relationships with doctors (RWD)	RWD1	0.841	0.8666	0.6841	0.867
	RWD2	0.811			
	RWD3	0.829			
Loss aversion (LA)	LA1	0.826	0.8975	0.6864	0.897
	LA2	0.83			
	LA3	0.827			
	LA4	0.831			
Performance risk (PR)	PR1	0.823	0.8549	0.6626	0.856
	PR2	0.807			
	PR3	0.812			
Medical AI resistance bias (AIRB)	AIRB1	0.802	0.8419	0.6398	0.842
	AIRB2	0.82			
	AIRB3	0.777			
Switching costs (SC)	SC1	0.828	0.7849	0.6462	0.784
	SC2	0.779			
Perceived personalization (PP)	PP1	0.794	0.7692	0.6249	0.783
	PP2	0.787			
Perceived anonymity (PA)	PA1	0.836	0.9052	0.7048	0.906
	PA2	0.847			
	PA3	0.837			
	PA4	0.838			
Flow experience (FE)	FE1	0.817	0.8606	0.673	0.889
	FE2	0.822			
	FE3	0.822			
	FE4	0.803			
Switching intention (SI)	SI1	0.828	0.8703	0.6911	0.869
	SI2	0.829			
	SI3	0.837			

**Table 4:** Square roots of AVE.

Variables	PP	PA	FE	HU	AIRB	RWD	PR	LA	SC	DSSM	HIO	CYB	SI
PP	0.791												
PA	0.412	0.84											
FE	0.489	0.578	0.82										
HU	−0.529	−0.566	−0.417	0.805									
AIRB	−0.352	−0.378	−0.278	0.638	0.8								
RWD	−0.297	−0.334	−0.241	0.311	0.297	0.827							
PR	−0.315	−0.383	−0.269	0.546	0.355	0.23	0.814						
LA	0.321	0.373	0.266	−0.493	−0.328	−0.277	−0.556	0.828					
SC	−0.067	−0.084	−0.058	0.124	0.08	0.043	0.271	−0.063	0.804				
DSSM	−0.267	−0.297	−0.215	0.411	0.271	0.209	0.271	−0.251	0.061	0.83			
HIO	−0.378	−0.407	−0.299	0.536	0.354	0.277	0.377	−0.32	0.088	0.393	0.826		
CYB	−0.459	−0.513	−0.372	0.718	0.473	0.365	0.468	−0.44	0.104	0.548	0.557	0.804	
SI	0.196	0.23	0.386	−0.199	−0.254	−0.104	−0.14	0.111	−0.154	0.119	−0.095	−0.108	0.831

To investigate whether the study variables differed across demographic groups, this research employed analysis of variance. The results showed that the switching cost (SC) differed significantly by prior experience with online health consultations (t = −2.386, *p* = 0.017), with respondents who had used online health consultation services reporting significantly lower SC levels than those who had not. No significant differences were found in the measured variables across other demographic dimensions. Multicollinearity diagnostics showed that tolerance values ranged from 0.415 to 0.856 and variance inflation factor (VIF) values ranged from 1.168 to 2.409, indicating no serious multicollinearity concern.

### 4.2 Hypothesis paths test

This study employs Structural Equation Modeling (SEM) and fuzzy-set Qualitative Comparative Analysis (fsQCA). First, SEM is used to test the hypothesized relationships within the PPM model. Second, fsQCA is applied to explore configurations of antecedent conditions associated with users' switching intentions in health information seeking. SEM is a variable-oriented research method that estimates net associations among variables and assesses overall model fit. This study integrates SEM and fsQCA methods. After assessing the hypothesized relationships, it further explores different configurational pathways to enhance a systematic understanding of the factors associated with users' intentions to shift their primary reliance for health information seeking from social media to conversational AI.

Harman's single-factor test extracted 13 factors with eigenvalues greater than 1, while the first factor explained only 32.40% of the total variance, indicating that common method bias was unlikely to be a serious concern. Structural equation modeling analysis was firstly conducted using AMOS 26. The model fit indices are as follows: χ^2^/df = 3.107 (< 5), GFI = 0.96 (> 0.9), AGFI = 0.954 (> 0.9), CFI = 0.975 (> 0.9), IFI = 0.975 (> 0.9), TLI = 0.973 (> 0.9), NFI = 0.964 (> 0.9), RMSEA = 0.027 (< 0.05), all indices met the recommended thresholds, indicating a good model fit.

All hypotheses were supported except H10. Among the push factors, health information overload (β = 0.128, *p* < 0.01) and cyberchondria (β = 0.528, *p* < 0.01) were positively associated with dissatisfaction with social media services. Dissatisfaction, in turn, was positively associated with switching intention (β = 0.259, *p* < 0.01).

Among the pull factors, perceived personalization (β = 0.330, *p* < 0.01) and perceived anonymity (β = 0.430, *p* < 0.01) were positively associated with flow experience, which was positively associated with switching intention (β = 0.390, *p* < 0.01). Among the mooring factors, health condition uncertainty (β = 0.612, *p* < 0.01) and relationships with doctors (β = 0.101, *p* < 0.01) were positively associated with medical AI resistance bias. Loss aversion (β = 0.123, *p* < 0.01) and performance risk (β = 0.325, *p* < 0.01) were positively associated with switching costs. Medical AI resistance bias (β = −0.223, *p* < 0.01) and switching costs (β = −0.137, *p* < 0.01) were negatively associated with switching intention. The interaction between dissatisfaction and medical AI resistance bias was negative (β = −0.0905, *p* < 0.01), supporting H7. The interaction involving flow experience and switching costs was positive (β = 0.0335, *p* < 0.01), contrary to the hypothesized negative direction; therefore, H10 was not supported. The statistically significant paths and their standardized coefficients are presented in Figure 2.

**Figure 2:**
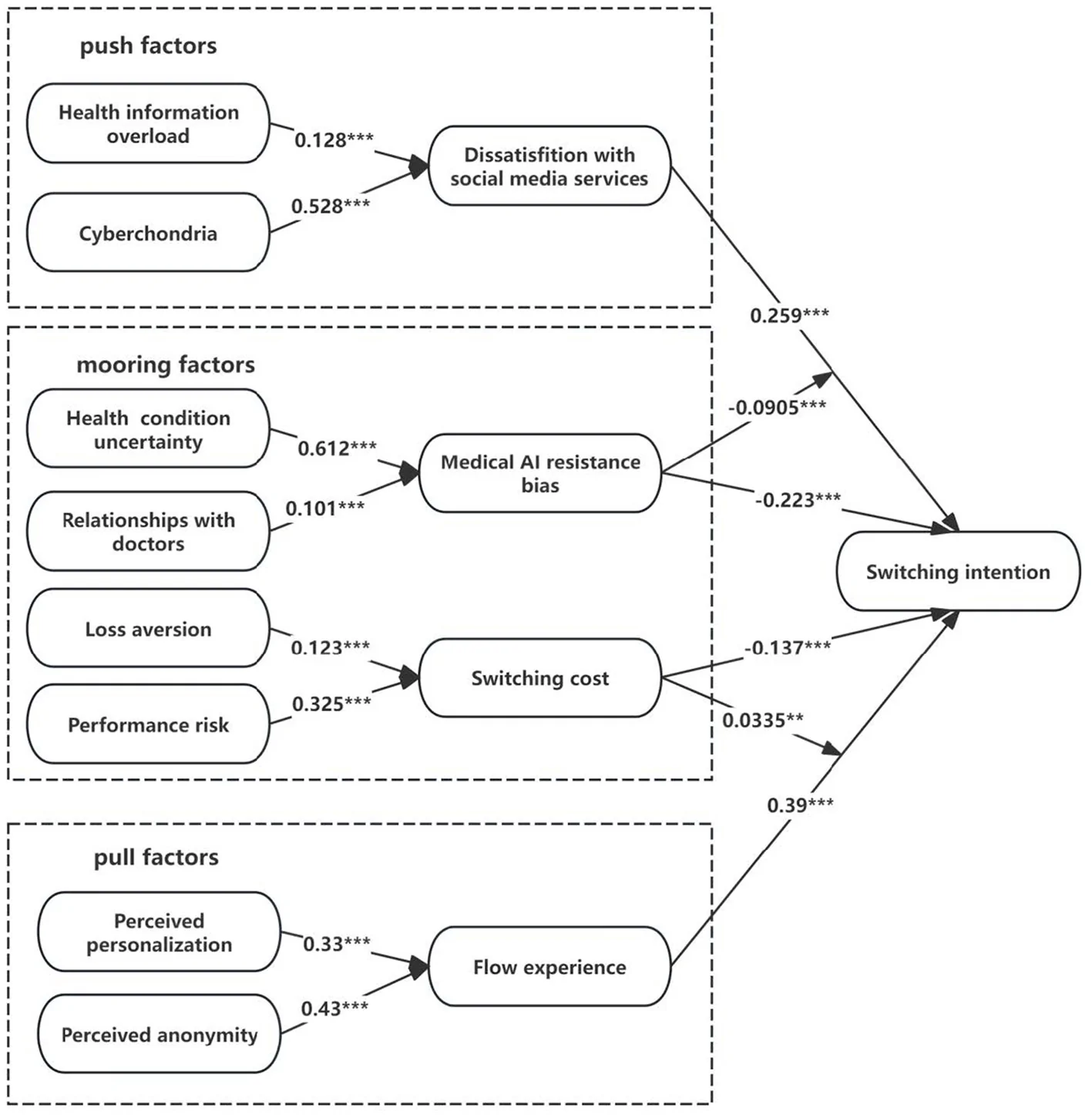
Statistical model of users' switching intention from social media to conversational AI for health information seeking. Only statistically significant paths are presented. ***p < 0.001, **p < 0.01, *p < 0.05.

### 4.3 Fuzzy-set Qualitative Comparative Analysis (fsQCA)

Greckhamer et al. (2018) noted that when constructing fsQCA models, in order to account for the maximum number of conditions that the sample size can accommodate and to ensure model parsimony, several lower-order conditions can be combined into theoretically meaningful higher-order concepts to enhance parsimony. Therefore, this study selects six variables—Health information overload (HIO), Cyberchondria (CYB), medical AI resistance bias (AIRB), switching costs (SC), perceived personalization (PP), and perceived anonymity (PA)—as antecedent conditions and constructs an fsQCA model with a single outcome variable (switching intention) to further examine which configurations of these conditions are associated with high or low intention to shift primary reliance for health information seeking from social media to conversational AI.

Following Fiss's (2011) calibration criteria, three anchors—full membership, the crossover point, and full non-membership—were specified. The 95th, 50th, and 5th percentiles were used to determine these three anchors, and the data were calibrated using fsQCA 4.0. The consistency values of all antecedent variables are below 0.9, indicating that no single antecedent variable constitutes a necessary condition for the outcome variable. The analysis therefore proceeds to the configurational analysis stage.

A truth table of 64 (2^6^) rows was first constructed, with each row representing a possible combination of the antecedent conditions. The frequency threshold was set to 15, the consistency threshold was set to 0.85, and the PRI threshold was set to 0.67 (Cartaxo et al., 2024). The results are shown in Table 5.

**Table 5:** fsQCA results.

Conditions	High Switching intention				Low Switching intention		
	S1	S2	S3	S4	H1	H2	H3
PP	•				×	•	×
PA		•	•	•	×	×	×
AIRB	×	×	×		•	•	•
SC	×	×	×	×	•	•	×
HIO	×	×		×	×	•	•
CYB			×	×			•
Consistency	0.838	0.838	0.844	0.833	0.852	0.862	0.862
Raw coverage	0.375	0.386	0.388	0.378	0.222	0.219	0.218
Unique coverage	0.036	0.010	0.040	0.030	0.06	0.053	0.061
Solution consistency	0.812				0.813		
Solution coverage	0.492				0.351		

• indicates that the core condition exists, × indicates that the core condition absent, × indicates that the peripheral condition absent and “blank” indicates that the condition is optional.

Based on the fsQCA approach, this study identifies four effective configurations (S1–S4) associated with high switching intention and three configurations (H1–H3) associated with low switching intention (Table 5). These seven configurations reflect different combinational pathways of antecedent conditions, which are analyzed separately below.

S1 suggests that “PP^*^~AIRB^*^ ~SC^*^~HIO” is associated with high switching intention. When perceived personalization is at a high level and both medical AI resistance bias and switching cost are low, users can still develop a strong switching intention even if they do not experience health information overload on social media.

S2 suggests that “PA^*^~AIRB^*^ ~SC^*^~HIO” is associated with high switching intention. When users develop a strong perception of privacy protection on the new platform (a pull factor), and this is likewise accompanied by low medical AI resistance bias and low switching cost, they still exhibit a strong intention to switch.

S3 suggests that “PA^*^~AIRB^*^ ~SC^*^~CYB” is associated with high switching intention. Similar to S2, S3 also takes perceived anonymity as the core condition, combined with low medical AI resistance bias and low switching costs, but it is characterized by a low level of cyberchondria. This indicates that even when users are not experiencing health anxiety, a strong switching intention can still emerge under a configuration characterized by anonymity and low mooring factors.

S4 suggests that “PA^*^ ~SC^*^~HIO^*^~CYB” is associated with high switching intention. This indicates that users are not simply being “pushed along” by the original context. Within this configuration, perceived anonymity and low switching costs are jointly associated with high switching intention, even when push factors are absent or low.

By comparing the four high-intention pathways, low switching costs appear repeatedly, suggesting that weak mooring is a recurrent feature of configurations associated with high switching intention rather than an individually necessary condition. Push factors are not necessary conditions. High switching intention can also emerge when push conditions are absent, provided that perceived pull factors and weak mooring jointly characterize the configuration.

H1 suggests that “~PP^*^~PA^*^ AIRB^*^ SC^*^~HIO” is associated with low switching intention. This indicates that when both pull and push factors are low, users are not willing to switch to AI-based conversational health information seeking due to high medical AI resistance bias and high switching costs.

H2 suggests that “PP^*^~PA^*^ AIRB^*^ SC^*^HIO” is associated with low switching intention. Unlike H1, users are in a context where high push factors (health information overload) and high pull factors (perceived personalization) coexist. However, when medical AI resistance bias and switching costs are both high, the configuration remains associated with low switching intention despite the simultaneous presence of push and pull conditions.

H3 suggests that “~PP^*^~PA^*^ AIRB^*^ ~SC^*^HIO^*^CYB” is associated with low switching intention. This indicates that in a high-push, low-pull context, users are dissatisfied with seeking health information on social media and face low switching costs, but do not perceive an experiential advantage of AI. Within this configuration, high medical AI resistance bias co-occurs with low switching intention. This configuration indicates only low switching intention toward conversational AI.

To assess the robustness of the fsQCA results, the case-frequency threshold of 15 and the PRI threshold of 0.67 were held constant, while the consistency threshold was increased from 0.85 to 0.88 (Schneider et al., 2010). After this adjustment, four configurations for high switching intention and three configurations for low switching intention were identified, consistent with the baseline results. The core conditions and their directions remained unchanged, while solution consistency and coverage varied only slightly. These findings therefore support the robustness of the configurational results.

## 5 Discussion

### 5.1 General discussion

The results of the SEM analysis indicate that users' dissatisfaction with social media services and their flow experience when using new technologies are positively associated with switching intention, whereas medical AI resistance bias and switching costs are negatively associated with switching intention. The fsQCA analysis further reveals multiple equifinal sufficient pathways and configurational asymmetry. Push factors are not necessary conditions for high switching intention. High switching intention may arise when users are dissatisfied with social media as a health-information-seeking channel or perceive conversational AI as an attractive alternative channel under conditions of weak mooring. Importantly, this reflects an intended shift in primary reliance for comparable health-information-seeking tasks, rather than a willingness to substitute conversational AI for professional medical care. In contrast, high medical AI resistance bias and switching costs recur as core conditions in particular configurations associated with low switching intention; neither condition is individually necessary.

Overall, high and low switching intention display asymmetric configurational patterns. SEM identifies dissatisfaction with social media and flow experience with conversational AI as the main variables positively associated with switching intention. The fsQCA results show that high switching intention is generally associated with AI personalization or anonymity combined with low switching costs, whereas low switching intention is mainly associated with strong medical AI resistance bias and high switching costs. Thus, push factors alone may be insufficient for high switching intention; configurations associated with high intention also feature attractive AI attributes and weak mooring conditions.

Regarding push factors, health information overload and cyberchondria are significantly associated with users' dissatisfaction, which in turn is associated with their intention to shift primary reliance between health-information-seeking channels. The CB-SEM results indicate that dissatisfaction with social media services is significantly positively associated with users' switching intentions. However, the fsQCA results further indicate that health information overload and cyberchondria appear as core absent or irrelevant conditions in configurations associated with high switching intention, whereas they can emerge as core present conditions in configurations associated with low switching intention. On social media, information overload can lead to information avoidance (Dai et al., 2020), while cyberchondria is likewise characterized by repetitive searching (Starcevic et al., 2021). In health contexts, this suggests that information-related stress is more likely to trigger avoidance rather than motivate the exploration of more complex new tools (Khaleel et al., 2020). Evidence from another AI-mediated decision context indicates that the way AI-generated summaries condense information can shape decision satisfaction (Wang et al., 2026). In health information seeking, information overload and cyberchondria may lead to intermittent discontinuance or information avoidance rather than the adoption of an alternative technology. Accordingly, push factors may be associated with withdrawal from social media without necessarily generating a stronger intention to rely on conversational AI as an alternative information channel. Reduced reliance on social media should therefore not be interpreted as a preference for AI or as a shift away from professional care.

Regarding pull factors, perceived anonymity and perceived personalization are significantly positively associated with users' flow experience, which in turn is positively associated with their intention to shift primary reliance toward conversational AI. Interactive AI features can improve the quality of human–machine communication (Baabdullah et al., 2022). LLM-supported recommendation systems further demonstrate that preference alignment can be combined with explainable recommendation paths (Zhu et al., 2026). However, personalization may heighten perceived vulnerability when users feel tracked (Ameen et al., 2022) and may reduce trust and expected benefits in sensitive health contexts (Bol et al., 2018). Moreover, perceived anonymity should not be equated with actual privacy, because anonymized tracking data may still enable cross-platform identity alignment and re-identification (Shi et al., 2026). Nevertheless, perceived anonymity emerged as a core condition in multiple configurations associated with high switching intention. This suggests that its primary role is psychological: by reducing identity-related concerns and the perceived cost of disclosing sensitive information, anonymity facilitates relaxation and flow. Overall, personalization mainly enhances the interaction experience, whereas anonymity reduces perceived identity risk; neither factor alone guarantees switching. Personalization, anonymity, and flow reflect perceived interaction quality rather than factual or clinical quality. Conversational AI may generate inaccurate or fabricated information, contributing to misinformation and potential overreliance (Denecke et al., 2024). It should therefore be treated as a complementary source rather than a substitute for professional judgment.

Regarding mooring factors, this study finds that they exhibit a configurational constraining role in the formation of switching intention. Health condition uncertainty and relationships with doctors are positively associated with medical AI resistance bias, while medical AI resistance bias is negatively associated with switching intention and attenuates the positive association between DIS and SI. In health-related contexts, individuals tend to manage uncertainty through approaches that are perceived as controllable, trustworthy, and accountable. While online information seeking can indeed serve as a means of uncertainty management, this does not imply that AI is trusted to the same extent (Rains, 2014). At the same time, related studies indicate that patients' trust in “AI involvement in diagnosis” remains significantly constrained (Chen and Cui, 2025). Medical AI resistance bias continues to be a stable phenomenon on the user side, and even when AI performs better, individuals still tend to maintain a low intention to switch. Meanwhile, loss aversion and performance risk are positively associated with perceived switching costs, which are negatively associated with switching intention, reflecting the constraining effects of status quo bias and risk avoidance on users' switching intentions. By aggregating the mooring factors into medical AI resistance bias and switching cost through fsQCA, the analysis further reveals that pathways associated with high switching intention generally share a common precondition of low mooring. In contrast, in configurations associated with low switching intention, medical AI resistance bias and switching costs repeatedly emerge as core conditions, even when push and pull factors coexist. This pattern is consistent with status quo bias in information-system migration, in which incumbent-system habits, switching costs, and inertia are associated with continued reliance on existing systems (Polites and Karahanna, 2012).

### 5.2 Theoretical contribution

This study offers several key theoretical contributions. First, by extending the push–pull–mooring (PPM) model to users' intention to shift primary reliance between social media and conversational AI as health-information-seeking channels, this study broadens the theoretical understanding of health information seeking and platform switching. Second, this study develops an integrative framework for examining switching intention between social media and conversational AI and clarifies the hypothesized associations among push, pull, and mooring factors. Specifically, health information overload and cyberchondria are positively associated with dissatisfaction with social media, and dissatisfaction is positively associated with switching intention. Perceived personalization and perceived anonymity are positively associated with flow experience, which is positively associated with switching intention. Third, this study identifies two critical mooring factors—medical AI resistance bias and switching costs—and reveals their moderating effects in SEM and their recurring constraining roles within configurations associated with low switching intention in fsQCA. Furthermore, by integrating structural equation modeling (SEM) with fuzzy-set qualitative comparative analysis (fsQCA), this study not only examines the hypothesized associations but also uncovers multiple equivalent configurational pathways, thereby enriching the theoretical understanding of switching intention in health information seeking.

### 5.3 Practical implications

This study offers practical implications for three stakeholders. Conversational AI providers should enhance personalization and anonymity, simplify interfaces and onboarding, and reduce perceived switching costs. For health-related queries, systems should cite credible sources, communicate uncertainty, request missing context, protect sensitive data, and direct users to professional care when symptoms are serious or decisions are high risk. ChatGPT Health provides a current example: it helps users understand health information and prepare for clinical appointments while being explicitly positioned as support rather than a substitute for diagnosis or treatment (OpenA, 2026). Social media platforms should mitigate information overload and cyberchondria by prioritizing verified health sources, labeling uncertain or promotional content, limiting repetitive recommendations, and linking users to professional services. Finally, health communication policymakers should establish standards for source attribution, privacy protection, uncertainty disclosure, human oversight, and referral procedures for high-risk queries. They should also promote public health and AI literacy. Together, these measures can position conversational AI as a complementary information tool while preserving professional medical judgment as the basis of diagnosis and treatment (World Health Organization, 2021).

### 5.4 Limitations and suggestions for future research

This study has three limitations. First, the online sample comprised users with prior AI-based health consultation experience; findings may not generalize beyond digitally engaged, AI-experienced populations. Second, the cross-sectional self-report design limits causal inference and cannot establish whether switching intention translates into subsequent channel use. Third, the study focused on general-purpose LLMs and did not assess output accuracy or clinical safety. Medical AI resistance bias and perceived anonymity do not directly capture perceived medical accuracy, trust, diagnostic competence, or broader privacy concerns—potentially relevant omissions highlighted by updated IS success, quality-dimensions, and RAIDUA frameworks. Future research should compare professional and general-purpose systems and combine these constructs with output audits and behavioral measures of verification and care seeking, given risks of inaccuracy and overreliance (Denecke et al., 2024).

## Data Availability

**T**he raw data supporting the conclusions of this article will be made available by the authors, without undue reservation.
